# Multiple Genome Sequences of Important Beer-Spoiling Lactic Acid Bacteria

**DOI:** 10.1128/genomeA.01077-16

**Published:** 2016-10-06

**Authors:** Andreas J. Geissler, Jürgen Behr, Rudi F. Vogel

**Affiliations:** a Technische Universität München, Lehrstuhl für Technische Mikrobiologie, Freising, Germany; b Technische Universität München, Bavarian Center for Biomolecular Mass Spectrometry, Freising, Germany

## Abstract

Seven strains of important beer-spoiling lactic acid bacteria were sequenced using single-molecule real-time sequencing. Complete genomes were obtained for strains of *Lactobacillus paracollinoides*, *Lactobacillus lindneri*, and *Pediococcus claussenii*. The analysis of these genomes emphasizes the role of plasmids as the genomic foundation of beer-spoiling ability.

## GENOME ANNOUNCEMENT

Beer is a harsh environment with which bacteria have to cope, as antibacterial hurdles, such as the low pH and the presence of antibacterial hops, have to be taken. Some lactic acid bacteria (LAB) are capable of growing in and spoiling beer (1, 2). To gain insights into the genomic adaptation of LAB to beer, we sequenced the complete genomes of seven different brewery isolates, comprising three different species.

Beer spoilage ability was tested as described previously (3). DNA isolation, sequencing, assembly, annotation, and genome analysis were done as described previously for *Lactobacillus backii* (4). High-molecular-weight DNA was purified from de Man-Rogosa-Sharpe (MRS) liquid cultures using the Genomic-tip 100/G kit (Qiagen). Single-molecule real-time sequencing (SMRT) (PacBio RS II) was carried out at GATC Biotech (Konstanz, Germany) (5). An insert size of 8 to 12 kb was selected for library creation, resulting in at least 200 Mb of raw data from one to two SMRT cells (1 × 120-min movies) applying P4-C2 chemistry. Assembly was done with SMRT Analysis version 2.2.0.p2, using the Hierarchical Genome Assembly Process (HGAP) (6), and completed by manual curation (https://github.com/PacificBiosciences/Bioinformatics-Training/wiki/Finishing-Bacterial-Genomes). Genomes were annotated using the NCBI Prokaryotic Genome Annotation Pipeline (PGAP) and Rapid Annotations using Subsystems Technology (RAST) (7–9). Genomes were further analyzed with CMG-BioTools, BADGE, and PSORTb (3, 10, 11).

Strain characteristics, sequencing statistics, genome information, and accession numbers are listed in Table 1. Chromosome sizes range from 1.39 Mbp for *Lactobacillus lindneri* to 3.48 Mbp for *Lactobacillus paracollinoides*, with G+C contents ranging from 34.3 to 47.2%. We found zero to eight plasmids with G+C contents from 34.7 to 44.5% and sizes from 13,353 bp to 57,063 bp. The analysis of RAST-annotated genomes resulted in the following core genomes: *Lactobacillus paracollinoides* with 2,503 gene families, *L. lindneri* with 1,305 gene families, and *Pediococcus claussenii* with 1,687 gene families, while *P. claussenii* ATCC BAA-344 was also included in the latter calculation (12). Chromosomes encode four complete rRNA operons in case of *P. claussenii*, five in case of *L. paracollinoides*, and six for *L. lindneri*.

**TABLE 1:** Strain characteristics, sequencing statistics, genome information, and accession numbers

Species	Strain	Source	Beer spoilage ability^a^	Biosample no.^b^	Accession no.^c^	Avg coverage of HGAP assembly (×)	Size (Mbp)	No. of contigs^d^	G+C content (%)	PEG^e^	CDSs^f^
*L. paracollinoides*	TMW 1.1979 (DSM 20197)	Beer	NB	SAMN04505735	CP014912−CP014914	107	3.32	3	47.0	2,953	2,872
*L. paracollinoides*	TMW 1.1994	Brewery environment	B	SAMN04505736	CP014915−CP014923	102	3.66	9	46.8	3,363	3,219
*L. paracollinoides*	TMW 1.1995	Pilsner beer	B	SAMN04505737	CP014924−CP014932	88	3.75	9	46.6	3,378	3,286
*L. lindneri*	TMW 1.481	Brewery environment	B	SAMN04505733	CP014907−CP014911	143	1.45	5	34.4	1,429	1,387
*L. lindneri*	TMW 1.1993	Beer	B	SAMN04505734	CP014872	262	1.39	1	34.3	1,347	1,305
*P. claussenii*	TMW 2.53	Brewery environment	NB-B	SAMN04505731	CP014933−CP014935	192	1.95	3	37.1	1,895	1,849
*P. claussenii*	TMW 2.54	Brewery environment	B	SAMN04505732	CP014936−CP014939	146	1.99	4	37.1	1,940	1,884

a NB, nonspoiler; B, spoiler; NB-B, unstable, mostly B.

b All BioSamples are part of BioProject PRJNA290141.

c Accession numbers are listed for all contigs of each whole genome (as range).

d In chromosome plus plasmids and partial plasmids.

e PEG, number of protein-encoding genes based on RAST annotation.

f CDSs, number of coding sequences (total) based on NCBI PGAP.

All seven genomes were compared to each other as well as to the genomes of 17 strains with relevance for beer spoiling, including one *P. claussenii*, five *L. backii*, five *P. damnosus*, and six *L. brevis* genomes (3, 4, 12–16). We found that the investigated LAB species with relevance for beer spoiling are characterized by different genomic preconditions regarding chromosome size, number of proteins, G+C content, coding density, codon usage, amino acid usage, proteome similarity, chromosome (DNA) similarity, functional pattern (SEED/COG), and subcellular localization of proteins. Regarding chromosomal properties, beer-spoiling LAB species cover the whole diversity within the genus *Lactobacillus*. In contrast, brewery isolates of the abovementioned species, although to a different extent, share a number of highly homologous plasmid-carried genes, including the important lifestyle markers *horA*, *horC*, and *fabZ* (3, 17, 18). This emphasizes the role of plasmids in beer-spoiling ability (3, 19, 20).

### Accession number(s).

The seven complete genomes have been deposited in DDBJ/EMBL/GenBank under the accession numbers listed in Table 1.
